# Supplementary material for: The m6A pathway protects the transcriptome integrity by restricting RNA chimera formation in plants
Source: Life Sci Alliance. 2019 May 29;2(3):e201900393. doi: 10.26508/lsa.201900393 (PMC6545605; doi:10.26508/lsa.201900393)
Supplement: Supplementary file 1 [file LSA-2019-00393_TableS1.doc]

**Table S1 : List of genes showing a specific increased of read counts in the 500bp region downstream from all expressed genes in *nerd-*1 mutant plants.**

| **Gene ID** | **Mean WT** | **Mean *nerd-1*** | **Mean *nerd-1*+T** | **FC_WT/*nerd-1*** | **FC_WT/*nerd-1*+T** |
| --- | --- | --- | --- | --- | --- |
| | AT1G05890 | | --- | | AT1G10350 | | AT1G11360 | | AT1G16700 | | AT1G20405 | | AT1G23935 | | AT1G26150 | | AT1G28320 | | AT1G30910 | | AT1G32940 | | AT1G44750 | | AT1G52310 | | AT1G65040 | | AT1G70290 | | AT1G70560 | | AT1G71340 | | AT1G78420 | | AT1G79500 | | AT1G79730 | | AT1G79790 | | AT2G02360 | | AT2G04700 | | AT2G11270 | | AT2G11890 | | AT2G17650 | | AT2G17780 | | AT2G18040 | | AT2G20480 | | AT2G20630 | | AT2G21440 | | AT2G27260 | | AT2G30370 | | AT2G31870 | | AT2G34450 | | AT2G36000 | | AT2G40316 | | AT2G44190 | | AT2G47760 | | AT2G47930 | | AT3G08610 | | AT3G09410 | | AT3G11910 | | AT3G13030 | | AT3G17225 | | AT3G17650 | | AT3G22960 | | AT3G23255 | | AT3G24080 | | AT3G26360 | | AT3G27430 | | AT3G42950 | | AT3G44200 | | AT3G44890 | | AT3G45443 | | AT3G47890 | | AT3G48590 | | AT3G48710 | | AT3G55200 | | AT3G58270 | | AT3G60740 | | AT3G63250 | | AT3G63340 | | AT4G02150 | | AT4G04670 | | AT4G07990 | | AT4G08110 | | AT4G08170 | | AT4G08540 | | AT4G12040 | | AT4G12610 | | AT4G18160 | | AT4G18400 | | AT4G19829 | | AT4G19830 | | AT4G27430 | | AT4G27760 | | AT4G28270 | | AT4G28860 | | AT4G30580 | | AT4G33300 | | AT4G34110 | | AT4G35250 | | AT5G03850 | | AT5G04220 | | AT5G05660 | | AT5G07630 | | AT5G08060 | | AT5G08650 | | AT5G16610 | | AT5G19900 | | AT5G20110 | | AT5G22340 | | AT5G24670 | | AT5G25475 | | AT5G27300 | | AT5G35100 | | AT5G36890 | | AT5G40930 | | AT5G41620 | | AT5G45190 | | AT5G46160 | | AT5G47760 | | AT5G48120 | | AT5G53890 | | AT5G54940 | | AT5G60600 | | AT5G65000 | | | 8,09E-07 | | --- | | 1,62E-06 | | 2,83E-06 | | 3,60E-07 | | 2,16E-06 | | 2,29E-06 | | 5,14E-07 | | 2,95E-07 | | 1,48E-07 | | 5,14E-07 | | 1,00E-06 | | 1,02E-06 | | 9,95E-06 | | 2,17E-06 | | 7,06E-08 | | 5,36E-06 | | 2,12E-07 | | 4,20E-06 | | 4,56E-07 | | 7,33E-06 | | 4,64E-06 | | 5,20E-07 | | 1,17E-06 | | 4,84E-06 | | 2,66E-06 | | 2,11E-06 | | 7,70E-08 | | 6,87E-07 | | 8,03E-07 | | 1,48E-07 | | 8,15E-07 | | 7,06E-08 | | 4,18E-06 | | 2,47E-05 | | 1,97E-06 | | 2,89E-07 | | 9,37E-07 | | 2,89E-07 | | 8,90E-06 | | 4,37E-07 | | 4,41E-06 | | 9,50E-07 | | 4,13E-06 | | 3,98E-06 | | 6,63E-06 | | 2,78E-06 | | 9,65E-06 | | 1,03E-05 | | 1,94E-06 | | 7,06E-08 | | 3,76E-06 | | 1,23E-06 | | 1,48E-07 | | 4,37E-07 | | 5,39E-07 | | 3,31E-06 | | 2,18E-07 | | 2,25E-07 | | 1,41E-07 | | 3,40E-06 | | 2,25E-06 | | 0 | | 5,20E-07 | | 1,48E-07 | | 4,56E-07 | | 2,16E-06 | | 6,03E-07 | | 1,41E-07 | | 2,67E-06 | | 2,36E-06 | | 2,89E-06 | | 1,48E-07 | | 7,13E-07 | | 1,09E-06 | | 1,12E-06 | | 2,31E-07 | | 4,56E-07 | | 1,58E-05 | | 6,02E-06 | | 1,57E-06 | | 5,20E-07 | | 7,06E-08 | | 2,95E-07 | | 4,30E-07 | | 1,33E-06 | | 4,22E-06 | | 1,48E-07 | | 7,70E-08 | | 8,22E-07 | | 9,01E-06 | | 7,70E-08 | | 1,48E-07 | | 1,66E-06 | | 7,96E-07 | | 1,41E-07 | | 3,66E-07 | | 8,99E-07 | | 4,24E-06 | | 2,70E-06 | | 2,24E-06 | | 1,98E-06 | | 1,10E-06 | | 2,95E-07 | | 9,96E-06 | | 1,90E-06 | | 1,83E-06 | | 8,22E-07 | | | 1,02E-05 | | --- | | 5,35E-06 | | 7,83E-06 | | 1,61E-06 | | 6,51E-06 | | 6,01E-06 | | 1,95E-06 | | 9,22E-07 | | 1,85E-06 | | 1,42E-06 | | 8,66E-06 | | 2,76E-06 | | 4,26E-05 | | 5,85E-06 | | 1,55E-07 | | 2,65E-05 | | 8,65E-07 | | 9,85E-06 | | 2,19E-06 | | 1,52E-05 | | 1,05E-05 | | 1,83E-06 | | 1,16E-05 | | 1,82E-05 | | 6,93E-06 | | 4,38E-06 | | 5,36E-07 | | 1,48E-06 | | 3,15E-06 | | 4,65E-07 | | 2,98E-06 | | 2,19E-07 | | 1,44E-05 | | 0,000158438 | | 7,56E-06 | | 1,20E-06 | | 2,49E-06 | | 1,39E-06 | | 2,02E-05 | | 1,01E-06 | | 1,87E-05 | | 1,96E-06 | | 2,20E-05 | | 8,34E-06 | | 1,70E-05 | | 1,13E-05 | | 1,67E-05 | | 2,23E-05 | | 3,66E-06 | | 3,45E-06 | | 1,12E-05 | | 3,08E-06 | | 8,31E-07 | | 4,20E-06 | | 1,36E-06 | | 1,08E-05 | | 8,10E-06 | | 0,000481753 | | 4,72E-07 | | 6,69E-06 | | 5,05E-06 | | 8,38E-07 | | 1,17E-06 | | 9,63E-07 | | 2,00E-06 | | 6,65E-06 | | 1,84E-06 | | 6,50E-07 | | 6,33E-06 | | 8,92E-06 | | 8,35E-06 | | 8,95E-07 | | 2,28E-06 | | 3,11E-06 | | 3,81E-06 | | 7,25E-07 | | 1,46E-06 | | 4,60E-05 | | 2,14E-05 | | 4,12E-06 | | 1,87E-06 | | 1,37E-06 | | 6,91E-07 | | 1,77E-06 | | 3,95E-06 | | 1,52E-05 | | 1,45E-06 | | 2,19E-07 | | 1,93E-06 | | 1,83E-05 | | 3,66E-07 | | 1,54E-06 | | 6,19E-06 | | 2,72E-06 | | 7,89E-07 | | 1,17E-06 | | 5,71E-06 | | 1,38E-05 | | 1,30E-05 | | 5,53E-06 | | 4,98E-06 | | 3,79E-06 | | 1,20E-06 | | 2,23E-05 | | 8,59E-06 | | 8,69E-06 | | 1,68E-06 | | | 1,44E-06 | | --- | | 1,10E-06 | | 3,85E-06 | | 1,24E-07 | | 0 | | 1,45E-06 | | 2,46E-07 | | 2,43E-07 | | 3,59E-07 | | 4,80E-07 | | 9,58E-07 | | 7,91E-07 | | 3,96E-06 | | 2,48E-06 | | 6,22E-08 | | 3,76E-06 | | 4,24E-07 | | 3,01E-06 | | 4,80E-07 | | 4,20E-06 | | 5,39E-06 | | 1,81E-07 | | 2,50E-06 | | 1,75E-06 | | 2,05E-06 | | 1,34E-06 | | 5,43E-07 | | 7,91E-07 | | 6,78E-07 | | 1,24E-07 | | 6,64E-07 | | 1,81E-07 | | 5,43E-06 | | 0,000124931 | | 1,98E-06 | | 1,24E-07 | | 1,81E-07 | | 6,05E-07 | | 8,04E-06 | | 6,22E-08 | | 5,49E-06 | | 3,65E-07 | | 1,12E-05 | | 3,01E-06 | | 4,32E-06 | | 9,34E-06 | | 8,20E-06 | | 1,19E-05 | | 1,24E-06 | | 1,81E-07 | | 3,58E-06 | | 1,81E-06 | | 4,15E-07 | | 6,10E-07 | | 1,84E-07 | | 3,45E-06 | | 7,26E-07 | | 1,24E-07 | | 6,16E-07 | | 5,31E-06 | | 1,27E-06 | | 0 | | 1,02E-06 | | 3,05E-07 | | 1,22E-06 | | 1,83E-06 | | 7,29E-07 | | 1,22E-07 | | 1,63E-06 | | 2,25E-06 | | 1,75E-06 | | 1,81E-07 | | 2,46E-07 | | 6,13E-07 | | 7,32E-07 | | 2,49E-07 | | 7,23E-07 | | 2,04E-05 | | 3,74E-06 | | 1,02E-06 | | 7,91E-07 | | 2,99E-07 | | 3,70E-07 | | 4,27E-07 | | 1,52E-06 | | 3,39E-06 | | 1,87E-07 | | 1,24E-07 | | 6,13E-07 | | 5,90E-06 | | 1,54E-06 | | 5,45E-07 | | 6,10E-07 | | 7,18E-07 | | 1,19E-07 | | 7,88E-07 | | 9,58E-07 | | 5,48E-06 | | 4,40E-06 | | 3,50E-06 | | 1,03E-06 | | 1,09E-06 | | 1,84E-07 | | 0,000113937 | | 4,23E-06 | | 0 | | 1,63E-06 | | | 12,58307195 | | --- | | 3,310042828 | | 2,765692707 | | 4,474191129 | | 3,019862914 | | 2,622975603 | | 3,787198993 | | 3,121196561 | | 12,50802303 | | 2,772323056 | | 8,645356281 | | 2,70812009 | | 4,279387254 | | 2,697962346 | | 2,192481721 | | 4,935931706 | | 4,082308938 | | 2,342407231 | | 4,807110374 | | 2,067363376 | | 2,262028688 | | 3,515080995 | | 9,968649442 | | 3,765259371 | | 2,599798572 | | 2,082629521 | | 6,962799894 | | 2,16165575 | | 3,92424499 | | 3,146618909 | | 3,659451884 | | 3,100445231 | | 3,434754201 | | 6,413604924 | | 3,838069729 | | 4,144108363 | | 2,655011369 | | 4,810043921 | | 2,269955326 | | 2,30969777 | | 4,237090497 | | 2,062887499 | | 5,318347676 | | 2,09512567 | | 2,561248591 | | 4,078680011 | | 1,727742687 | | 2,158215173 | | 1,887719825 | | 48,91172371 | | 2,98980618 | | 2,499937831 | | 5,627885636 | | 9,620057704 | | 2,514564949 | | 3,267408802 | | 37,09179736 | | 2144,288332 | | 3,341863462 | | 1,964887261 | | 2,245923109 | | Inf | | 2,251533337 | | 6,524224516 | | 4,392435709 | | 3,085260562 | | 3,048434105 | | 4,597526966 | | 2,370293637 | | 3,77592258 | | 2,889304672 | | 6,06225112 | | 3,195180468 | | 2,847890649 | | 3,415012196 | | 3,138832171 | | 3,19924902 | | 2,909432166 | | 3,551636591 | | 2,630468387 | | 3,595110248 | | 19,46206255 | | 2,340351029 | | 4,116996907 | | 2,97309768 | | 3,601460313 | | 9,795769602 | | 2,843606429 | | 2,344027623 | | 2,031687148 | | 4,756986983 | | 10,46112178 | | 3,720579726 | | 3,420077349 | | 5,587486064 | | 3,199251682 | | 6,358068631 | | 3,240807684 | | 4,82551549 | | 2,467937615 | | 2,517335502 | | 3,454035782 | | 4,07999853 | | 2,241588177 | | 4,518574884 | | 4,748950952 | | 2,050078441 | | | 1,784911436 | | --- | | 0,679509855 | | 1,360645157 | | 0,345895378 | | 0 | | 0,632486249 | | 0,478744772 | | 0,822944013 | | 2,430080952 | | 0,93520853 | | 0,956179134 | | 0,775115483 | | 0,398485274 | | 1,143462107 | | 0,88035934 | | 0,702148388 | | 2,000170306 | | 0,716985482 | | 1,053980426 | | 0,573185825 | | 1,161943146 | | 0,347775618 | | 2,140392089 | | 0,362502023 | | 0,771068259 | | 0,637560265 | | 7,044002487 | | 1,152129777 | | 0,845234466 | | 0,842319556 | | 0,814460471 | | 2,560075789 | | 1,298908343 | | 5,057221922 | | 1,006329609 | | 0,430459728 | | 0,192908043 | | 2,09288847 | | 0,903458729 | | 0,142438148 | | 1,245440079 | | 0,383645798 | | 2,700498981 | | 0,755489472 | | 0,651734643 | | 3,358501861 | | 0,850223195 | | 1,149701278 | | 0,637113926 | | 2,560075789 | | 0,952460322 | | 1,469277773 | | 2,812489643 | | 1,398169889 | | 0,340735266 | | 1,041520873 | | 3,326774987 | | 0,553573186 | | 4,361295585 | | 1,559382471 | | 0,56342477 | | NA | | 1,961558656 | | 2,067047804 | | 2,678894812 | | 0,846352402 | | 1,208310837 | | 0,860108782 | | 0,609438185 | | 0,953345472 | | 0,607429709 | | 1,224728248 | | 0,344977734 | | 0,561889114 | | 0,655275804 | | 1,076574617 | | 1,587248317 | | 1,288053438 | | 0,621293516 | | 0,651189563 | | 1,521699431 | | 4,239792239 | | 1,253791563 | | 0,991903635 | | 1,144054215 | | 0,802744385 | | 1,263479335 | | 1,614861926 | | 0,746380939 | | 0,654838964 | | 20,02740826 | | 3,693560286 | | 0,367173062 | | 0,901369259 | | 0,839858225 | | 2,154397361 | | 1,06576209 | | 1,291833328 | | 1,628995691 | | 1,561408434 | | 0,520159274 | | 0,996141346 | | 0,622051896 | | 11,43512917 | | 2,225890319 | | 0 | | 1,980758761 | |
