# Supplementary material for: The m6A pathway protects the transcriptome integrity by restricting RNA chimera formation in plants
Source: Life Sci Alliance. 2019 May 29;2(3):e201900393. doi: 10.26508/lsa.201900393 (PMC6545605; doi:10.26508/lsa.201900393)
Supplement: Supplementary file 2 [file LSA-2019-00393_TableS2.doc]

**Supplementary Table S2 : List of putative chimeric/extended mRNAs in nerd-1 plants.**

Fold change of GENE2 expression in plants depleted of FIP37 or MTA proteins are indicated based on data published in Shen et al, 2012 (1) and Anderson et al, 2018 (2). Yes indicates the presence of an high confidence m6A peak in GENE1 mRNA, based on data published by Shen et al, 2012 (1); Anderson et al, 2018 (2) ; Luo et al, 2014 (3). Abreviation : Chimeric/Chim ; Extended/Ext ; GENE1 only/G1only ; Tandem Gene/TG ; Not applicable/NA ; High-confidence/HC.

| GENE1 | GENE2 | TYPE | Evo. Cat. | GENE2 log2 FC fip37L/WT (1) | GENE2 log2 FC  MTA/WT (2) | HC m6A peak in GENE1 mRNA  (1)(2)(3) |
| --- | --- | --- | --- | --- | --- | --- |
| | AT1G05890 | | --- | | AT1G16700 | | AT1G23935 | | AT1G26150 | | AT1G32370 | | AT1G32940 | | AT1G71340 | | AT2G02360 | | AT2G11270 | | AT2G11890 | | AT2G17650 | | AT2G20480 | | AT2G20630 | | AT2G21440 | | AT2G44190 | | AT3G09410 | | AT3G13030 | | AT3G14180 | | AT3G17650 | | AT3G23255 | | AT3G28760 | | AT3G44890 | | AT3G45443 | | AT3G47890 | | AT3G63340 | | AT4G00690 | | AT4G02150 | | AT4G19830 | | AT4G30580 | | AT5G04220 | | AT5G07630 | | AT5G25475 | | AT5G47660 | | AT5G60600 | | AT5G65000 | | AT2G31870 | | AT1G11360 | | AT1G30910 | | AT1G44750 | | AT1G70290 | | AT1G78420 | | AT1G79500 | | AT1G79730 | | AT1G79790 | | AT2G04700 | | AT2G17780 | | AT2G27260 | | AT2G40316 | | AT4G12610 | | AT3G11910 | | AT3G60740 | | AT5G19900 | | AT5G24670 | | AT5G36890 | | | AT1G05894 | | --- | | AT1G16705 | | AT1G23940 | | AT1G26140 | | AT1G32375 | | AT1G32950 | | AT1G71330 | | AT2G02350 | | AT2G11280 | | AT2G11891 | | AT2G17660 | | AT2G20470 | | AT2G20625 | | AT2G21430 | | AT2G44195 | | AT3G09405 | | AT3G13020 | | AT3G14172 | | AT3G17660 | | AT3G23260 | | AT3G28750 | | AT3G44900 | | AT3G45440 | | AT3G47875 | | AT3G63320 | | AT4G00695 | | AT4G02140 | | AT4G19829 | | AT4G30570 | | AT5G04210 | | AT5G07640 | | AT5G25470 | | AT5G47670 | | AT5G60610 | | AT5G64990 | | AT2G31865 | | AT1G11350 | | NA | | NA | | NA | | NA | | NA | | NA | | NA | | NA | | NA | | NA | | NA | | NA | | NA | | NA | | NA | | NA | | NA | | | Chim. | | --- | | Chim. | | Chim. | | Chim. | | Chim. | | Chim. | | Chim. | | Chim. | | Chim. | | Chim. | | Chim. | | Chim. | | Chim. | | Chim. | | Chim. | | Chim. | | Chim. | | Chim. | | Chim. | | Chim | | Chim | | Chim | | Chim | | Chim | | Chim | | Chim | | Chim | | Chim | | Chim | | Chim | | Chim. | | Chim. | | Chim. | | Chim. | | Chim. | | Chim. | | Chim. | | Ext. | | Ext. | | Ext. | | Ext. | | Ext. | | Ext. | | Ext. | | Ext. | | Ext. | | Ext. | | Ext. | | Ext. | | Ext. | | Ext. | | Ext. | | Ext. | | Ext. | | | G1only | | --- | | TG | | Adjacent | | G1only | | NA | | TG | | Distant | | TG | | NA | | NA | | NA | | NA | | G1only | | Distant | | Adjacent | | TG | | TG | | G2 single | | G1only | | Adjacent | | TG | | NA | | TG | | TG | | TG | | G1 only | | NA | | NA | | Distant | | G1only | | G1only | | TG | | Adjacent | | G1 only | | G1 only | | TG | | Distant | | NA | | NA | | NA | | NA | | NA | | NA | | NA | | NA | | NA | | NA | | NA | | NA | | NA | | NA | | NA | | NA | | NA | | | 4,51196 | | --- | |  | | 1,73487 | | Inf | | 2,83475 | | 4,36296 | | 1,96378 | |  | |  | |  | |  | |  | |  | | 1,84582 | |  | | 1,97264 | | 1,37455 | |  | |  | | 0,967937 | | 1,26724 | | 3,17522 | | 2,55161 | |  | |  | | 1,98291 | |  | |  | | 1,3002 | |  | | 3,89197 | | Inf | | Inf | | 3,63779 | |  | | 2,15831 | |  | | NA | | NA | | NA | | NA | | NA | | NA | | NA | | NA | | NA | | NA | | NA | | NA | | NA | | NA | | NA | | NA | | NA | | | 2,435629265 | | --- | | 2,004796161 | |  | |  | | 2,236126958 | | 2,097817534 | | 0,956446936 | | 1,302030449 | | 1,820171909 | |  | |  | | 1,775808975 | |  | | 2,213311628 | |  | |  | |  | |  | | 0,78494535 | |  | |  | | 1,108959869 | | 2,390869422 | | 2,247487283 | | 0,704995317 | | 1,253987754 | | 1,070123948 | |  | | 0,75852226 | |  | | 1,951406839 | |  | |  | | 3,886896689 | | 2,000563581 | |  | |  | | NA | | NA | | NA | | NA | | NA | | NA | | NA | | NA | | NA | | NA | | NA | | NA | | NA | | NA | | NA | | NA | | NA | | | Yes (1,3) | | --- | | Yes (1,3) | |  | | Yes (1,3) | |  | | Yes (2) | | Yes (3) | | Yes (3) | |  | | Yes (3) | |  | |  | | Yes (1,3) | | Yes (1,3) | |  | | Yes (1,3) | |  | |  | | Yes (2) | | Yes (3) | | Yes (1) | | Yes (1,3) | |  | | Yes (1,3) | |  | |  | | Yes (3) | |  | | Yes (1) | |  | | Yes (1,3) | |  | | Yes (3) | | Yes (1) | | Yes (2) | |  | | Yes (1) | | Yes (1,3) | | Yes (3) | |  | | Yes (1) | | Yes (1) | | Yes (1,3) | | Yes (3) | | Yes (1,3) | |  | | Yes (3) | | Yes (1) | | Yes (3) | | Yes (1,3) | | Yes (1,3) | |  | | Yes (3) | | Yes (1,3) | |
