# Supplementary material for: The m6A pathway protects the transcriptome integrity by restricting RNA chimera formation in plants
Source: Life Sci Alliance. 2019 May 29;2(3):e201900393. doi: 10.26508/lsa.201900393 (PMC6545605; doi:10.26508/lsa.201900393)
Supplement: Supplementary file 3 [file LSA-2019-00393_TableS3.doc]

**Table S3 : Duplication classes of GENE1/GENE2 in *A. thaliana* and their characterized orthologs in *A. lyrata* and *C. rubella* status.**

| GENE1 | Status | Duplication type | A. lyrata ortholog | C. rubella ortholog | GENE2 | Status | Duplication type | A. lyrata ortholog | C. rubella ortholog |
| --- | --- | --- | --- | --- | --- | --- | --- | --- | --- |
| | AT1G05890 | | --- | | AT1G11360 | | AT1G16700 | | AT1G23935 | | AT1G26150 | | AT1G32370 | | AT1G32940 | | AT1G56460 | | AT1G71340 | | AT2G02360 | | AT2G11270 | | AT2G11890 | | AT2G17650 | | AT2G20480 | | AT2G20630 | | AT2G21440 | | AT2G24120 | | AT2G31870 | | AT2G44190 | | AT3G09410 | | AT3G13030 | | AT3G14180 | | AT3G17650 | | AT3G23255 | | AT3G26360 | | AT3G45443 | | AT3G47890 | | AT3G62460 | | AT3G63340 | | AT4G00690 | | AT4G02150 | | AT4G19830 | | AT4G30580 | | AT5G04220 | | AT5G07630 | | AT5G25475 | | AT5G47660 | | AT5G60600 | | AT5G65000 | | | dup | | --- | | dup | | dup | | dup | | dup | | single | | dup | | dup | | single | | dup | | dup | | single | | dup | | single | | dup | | dup | | dup | | dup | | dup | | dup | | dup | | dup | | dup | | single | | single | | single | | dup | | dup | | dup | | dup | | dup | | dup | | dup | | dup | | single | | dup | | dup | | single | | dup | | | tandem | | --- | | WGD/Segmental | | WGD/Segmental | | translocated_after_cr | | NA | | NA | | tandem | | NA | | single | | tandem | | translocated_after_al | | single | | NA | | NA | | WGD/Segmental | | NA | | NA | | tandem | | WGD/Segmental | | tandem | | tandem | | WGD/Segmental | | WGD/Segmental | | single | | single | | single | | tandem | | proxmial | | tandem | | translocated_after_al | | WGD/Segmental | | NA | | NA | | NA | | single | | tandem | | NA | | single | | NA | | | 470601 | | --- | | NA | | 471866 | | 313246 | | 472802 | | 336321 | | 473476 | | 474809 | | 316118 | | NA | | NA | | 931036 | | 480657 | | 906860 | | 491931 | | 481009 | | 481268 | | 482111 | | 322085 | | 478262 | | NA | | 478832 | | 473974/479246 | | 479840 | | 484399 | | NA | | 485153 | | 349310 | | 486842 | | NA | | 490357 | | 492887 | | 491693 | | 325069 | | 325378 | | 489405 | | 915270 | | 950808 | | 496721 | | | Carubv10012456m | | --- | | Carubv10009983m | | Carubv10010241m | | NA | | Carubv10008405m | | Carubv10012275m | | Carubv10008369m | | Carubv10021702m | | Carubv10021692m | | NA | | NA | | Carubv10014647m | | Carubv10011463m/Carubv10013267m | | Carubv10015012m | | Carubv10014347m | | Carubv10024686m | | Carubv10025273m | | Carubv10022947m | | Carubv10023133m | | Carubv10013780m | | Carubv10015366m | | Carubv10015321m | | Carubv10008440m/Carubv10013074m | | Carubv10014537m | | Carubv10018299m | | NA | | Carubv10016569m | | NA | | Carubv10018471m | | NA | | Carubv10003501m | | Carubv10005678m | | Carubv10005118m | | Carubv10003794m | | Carubv10002674m | | Carubv10003492m | | Carubv10027995m | | Carubv10025946m | | Carubv10026670m | | | AT1G05894 | | --- | | AT1G11350 | | AT1G16705 | | AT1G23940 | | AT1G26140 | | AT1G32375 | | AT1G32950 | | AT1G56470 | | AT1G71330 | | AT2G02350 | | AT2G11280 | | AT2G11891 | | AT2G17660 | | AT2G20470 | | AT2G20625 | | AT2G21430 | | AT2G24110 | | AT2G31865 | | AT2G44195 | | AT3G09405 | | AT3G13020 | | AT3G14172 | | AT3G17660 | | AT3G23260 | | AT3G26350 | | AT3G45440 | | AT3G47875 | | AT3G62455 | | AT3G63320 | | AT4G00695 | | AT4G02140 | | AT4G19829 | | AT4G30570 | | AT5G04210 | | AT5G07640 | | AT5G25470 | | AT5G47670 | | AT5G60610 | | AT5G64990 | | | dup | | --- | | dup | | dup | | dup | | dup | | dup | | dup | | pseudo | | dup | | dup | | pseudo | | single | | dup | | dup | | dup | | dup | | dup | | dup | | dup | | dup | | dup | | dup | | dup | | dup | | dup | | dup | | TE | | TE | | dup | | dup | | dup | | single | | dup | | dup | | dup | | dup | | dup | | dup | | dup | | | WGD/Segmental | | --- | | WGD/Segmental;Proximal | | tandem | | proximal | | WGD/Segmental | | NA | | tandem | |  | | translocated after al | | tandem | |  | | single | | WGD/Segmental | | NA | | tandem | | WGD/Segmental | | NA | | tandem | | proximal | | tandem | | tandem | | WGD/Segmental | | NA | | translocated after cr | | WGD/Segmental | | tandem | |  | |  | | tandem | | translocated after al | | NA | | NA | | NA | | translocated after al | | translocated after al | | tandem | | proximal | | translocated after al | | WGD/Segmental | | | 470603 | | --- | | 471271 | | 312643 | | 472652 | | 313365 | | NA | | 336380 | | NA | | NA | | 322564 | | NA | | NA | | 900113 | | 906859 | | NA | | 481008 | | NA | | 482111 | | 935348 | | 478260 | | NA | | 478831 | | 318525 | | 319045 | | 484398 | | 937207 | | NA | | NA | | 486840 | | NA | | AT1G02700 | | NA | | 944932 | | 337999 | | NA | | 489405 | | 494206 | | NA | | 496719 | | | Carubv10011325m | | --- | | NA | | Carubv10010638m | | Carubv10008275m | | Carubv10010845m | | Carubv10011317m | | NA | | NA | | NA | | Carubv10018877m | | NA | | NA | | Carubv10015429m | | Carubv10015460m | | NA | | Carubv10023719m | | NA | | Carubv10024816m | | Carubv10024890m | | Carubv10013780m | | Carubv10015366m | | Carubv10012830m | | NA | | Carubv10016106m | | Carubv10018851m | | Carubv10016788m | | NA | | NA | | Carubv10018471m | | NA | | Carubv10003114m | | NA | | Carubv10005214m | | NA | | NA | | Carubv10003492m | | Carubv10028250m | | NA | | Carubv10027144m | |

Statistic

| TYPE | Total genes | GENE1 | GENE2 | % Total genes | % GENE1 | % GENE2 | Gene 1 Fisher p-value | Gene 2 Fisher p-value | Gene 1 Fisher p-value | Gene 2 Fisher p-value |
| --- | --- | --- | --- | --- | --- | --- | --- | --- | --- | --- |
| | single | | --- | | WGD/Segmental | | tandem | | translocated duplicates | | Other duplicates | | Total | | | 3750 | | --- | | 5099 | | 1446 | | 987 | | 15939 | | 27221 | | | 9 | | --- | | 7 | | 10 | | 3 | | 10 | | 39 | | | 2 | | --- | | 8 | | 13 | | 6 | | 6 | | 35 | | | 13,9774125 | | --- | | 18,73186143 | | 5,312075236 | | 3,62587708 | | 58,55405753 | | | 23,07692308 | | --- | | 17,94871795 | | 25,64102564 | | 7,692307692 | | 25,64102564 | | | 5,714285714 | | --- | | 22,85714286 | | 37,14285714 | | 17,14285714 | | 17,14285714 | |  | | | 0,1791 | | --- | | 1 | | 0,0001461 | | 0,1816 | | 0,01722 | |  | | | 0,3112 | | --- | | 0,536 | | 5,19E-07 | | 7,73E-09 | | 0,003025 | |  | | | ns | | --- | | ns | | *** | | ns | | * | |  | | | ns | | --- | | ns | | *** | | *** | | ** | |
